# Recent Advances in Basic Research for Brain Arteriovenous Malformation

**DOI:** 10.3390/ijms20215324

**Published:** 2019-10-25

**Authors:** Leandro Barbosa Do Prado, Chul Han, S. Paul Oh, Hua Su

**Affiliations:** 1 Center for Cerebrovascular Research, Department of Anesthesia, University of California, San Francisco, CA 94143, USA; Leandro.BarbosaDoPrado@ucsf.edu; 2 Barrow Aneurysm & AVM Research Center, Barrow Neurological Institute/Dignity Health, Phoenix, AZ 85013, USA; Chul.Han@dignityhealth.org (C.H.); ohp@barrowneuro.org (S.P.O.)

**Keywords:** brain arteriovenous malformation, somatic mutation, RAS-mitogen-activated protein kinases (MAPK), hereditary hemorrhagic telangiectasia, TGFβ, PDGF-B/PDGFR-B

## Abstract

Arteriovenous malformations (AVMs) are abnormal connections of vessels that shunt blood directly from arteries into veins. Rupture of brain AVMs (bAVMs) can cause life-threatening intracranial bleeding. Even though the majority of bAVM cases are sporadic without a family history, some cases are familial. Most of the familial cases of bAVMs are associated with a genetic disorder called hereditary hemorrhagic telangiectasia (HHT). The mechanism of bAVM formation is not fully understood. The most important advances in bAVM basic science research is the identification of somatic mutations of genes in RAS-MAPK pathways. However, the mechanisms by which mutations of these genes lead to AVM formation are largely unknown. In this review, we summarized the latest advance in bAVM studies and discussed some pathways that play important roles in bAVM pathogenesis. We also discussed the therapeutic implications of these pathways.

## 1. Introduction

Brain arteriovenous malformations (AVMs) are abnormal vessels that are prone to rupture, causing life-threatening intracranial hemorrhage (ICH) [1]. The estimated prevalence of brain AVMs (bAVMs) is 0.05% (95% CI: 0.01% to 0.10%) among otherwise healthy individuals [2]. Patients with bAVMs may remain asymptomatic or experience epileptic seizures, focal neurological deficits, or ICH, which is the most feared complication and the primary reason to treat. Overall, bAVMs account for 25% of hemorrhagic strokes in adults younger than 50 years of age [3], and up to 40% of bAVM patients die or remain functionally impaired within one year after ICH [4]. Each of the existing treatment modalities carries a non-trivial rate of procedure-related complications, although the mortality varies among hospitals [5,6,7]. With the advance of imaging techniques, more asymptomatic bAVM patients will be diagnosed. The treatment of asymptomatic patients has become increasingly controversial because findings from a randomized trial of unruptured bAVM (ARUBA) showed that stroke and mortality were lower in unruptured bAVM patients randomized to conservative management than patients who received any interventional therapy [5,6,8,9,10,11]. Currently, there is no specific medical treatment available. Uncovering bAVM pathogenesis is essential for the development of specific therapies to minimize the need for invasive procedures.

More than 95% of bAVMs are sporadic cases without a clear family history. The causative gene for sporadic bAVM is largely unknown. Recent studies identified somatic mutations of genes in the RAS-MAPK pathways in sporadic bAVMs and extra-neural AVMs [12,13]. The significance of these genes in AVM formation needs to be studied further.

The familial forms of the more common sporadic disorders have been used to study the disease mechanisms of sporadic cerebrovascular diseases [14,15,16,17,18,19,20,21,22]. The most studied familial form of bAVM cases is associated with a genetic disorder called hereditary hemorrhagic telangiectasia (HHT). More than 90% of HHT patients have mutations in transforming growth factor β (TGFβ) family receptors, endoglin (*ENG*), or activin receptor-like kinase 1 (*ALK1* or *ACVLR1*).

Although many pathways and modifier genes are implicated in bAVM pathogenesis, in this review, we focused on recent discoveries in RAS-MAPK-ERK, TGFβ, platelet-derived growth factor b (Pdgfb) pathways, and non-cording RNAs in bAVM studies.

## 2. RAS-MAPK-ERK Signaling in Sporadic bAVM

The RAS pathway includes different signaling cascades, such as RAF-MEK-MAPK/ERK. This pathway regulates several critical cellular functions, including proliferation, growth, survival, and senescence [23]. It has been documented that alteration of the RAS-MAPK pathway triggers tumorigenesis in humans as a result of abnormal activation of receptor tyrosine kinases mutations [24]. Subsequent studies showed KRAS mutations are present in colorectal, lung, and biliary tract carcinogenesis, while mutations in NRAS and HRAS are frequently present in a high percentage in melanomas and salivary gland tumors, respectively [25]. Of note, 90% of pancreatic adenocarcinomas harbor a RAS mutation [26]. Furthermore, it has been shown that ERK5 regulates several signaling pathways involved in angiogenesis, whereby it can inhibit the expression of VEGF during hypoxic response [27].

Recent studies have identified somatic mutations in genes in the RAS-MAPK pathway in sporadic bAVMs and extra-neural AVMs [12,13], through next-generation sequencing of DNAs isolated from patients’ AVM lesions. The authors found somatic activating KRAS mutations in bAVM lesions from 45 of the 72 patients and in none of the 21 paired blood samples. The mutations included KRAS p.Gly12Val and the KRAS p.Gly12Asp mutations. Further investigation of the downstream signaling pathways, they found MAPK-ERK and PI3K-AKT pathways are activated by KRAS-activating mutations, in endothelial cell-enriched cultures derived from human bAVMs. The levels of ERK1/2 phosphorylation were increased in endothelial cells derived from bAVMs compared to the endothelial cells derived from normal brain vessels. AKT phosphorylation was not increased in bAVM endothelial cells. They showed that the mutations increased the expression of genes related to angiogenesis and Notch signaling. Introducing KRAS p.Gly12Val to cultured endothelial cells enhanced their migratory behavior. Interestingly, inhibition of MAPK-ERK but not PI3K pathway reversed the VEGF gene signature in endothelial cells [12]. A subsequent study showed that the prevalence of *KRAS/BRAF* mutation was 81% in bAVMs and 100% in spinal AVMs [28]. These data indicate that somatic mutations in KRAS may contribute to the pathogenesis of human bAVMs.

Somatic mutations in genes involved in the RAS/MAPK pathway have also been detected in peripheral vascular malformations. Mosaic variants in genes in the RAS/MAPK pathway, including KRAS, NRAS, BRAF, and MAP2K1, have been detected in the lesions of intracranial and extracranial sporadic vascular malformations in children [29]. The mutations are more frequent in high-flow (AVM) than in low-flow (cerebral cavernous malformation) lesions. Introduction of these mutations to zebrafish resulted in vascular malformations that recapitulate human phenotypes. Treatment with a BRAF inhibitor, Vemurafenib, restored blood flow in malformed vessels in zebrafish. Couto et al. detected somatic MAP2K1 mutations in 64% of extracranial AVMs [13]. The mutation alleles were enriched in endothelial cells.

## 3. TGF-β Signaling in Familiar bAVM

About 5% of bAVMs are linked to a genetic disorder, HHT, which is an autosomal dominant vascular disease that affects approximately 1 in 5000 people worldwide [30,31,32]. The major clinical feature of HHT is hemorrhage from AVMs in multiple organs, including the brain [33]. Three genes have been identified to cause HHT: *ENG* [34], *ALK1* or *ACVRL1* [35], and *SMAD4* [36]. HHT is classified into HHT1, HHT2, and JP (juvenile polyposis)-HHT, depending on the causative gene mutations. HHT1 (*ENG* mutations) and HHT2 (*ALK1* mutations) cover over 90% of all HHT cases [37]. Although clinical presentations are indistinguishable between HHT1 and HHT2, genotype-phenotype correlation studies have shown that HHT1 has a higher prevalence of AVMs in the brain and lungs, while HHT2 has a higher prevalence of AVMs in the liver and gastrointestinal tract [31,38,39,40,41]. Brain AVMs are present in 10.4% of patients with HHT. HHT1 patients have a significantly higher bAVM prevalence (13.4%) compared with HHT2 patients (2.4%) [42]. Most of the HHT-associated bAVMs are small (less than 3 cm) and have a Spetzler–Martin grade of 2 or less; whereas, in the sporadic bAVM population, the mean bAVM nidus size is about 3 cm, and the median Spetzler–Martin score is 3. While about 20% of these HHT-associated bAVMs present with rupture, nearly 50% of bAVMs are asymptomatic [42].

All identified genes associated with HHT are components of signal transduction of TGF-β family members [43]; thus, HHT has been considered a disease caused by defects in the signaling of TGF-β family member(s). However, detailed knowledge about the identity of the ligand(s), type II receptor(s), and downstream effectors genes of ENG-ALK1 signaling pertinent to AVM development are mostly unclear. Recent studies have shown that blockages for both BMP9 and BMP10 could induce AVM development in the retinal vasculature [44,45], but it is not entirely clear whether both BMP9 and BMP10 are needed for ENG-ALK1 signaling [46,47].

In addition to the canonical SMAD pathway, TGFβ family ligands also signal through non-SMAD signaling pathways [48]. ALK1-mutation increased pERK in cells treated with VEGF [49,50]. The phosphatase and tensin homolog (PTEN) connected BMP-9 activation of ALK1 to PI3K signaling in ECs [49,51]. BMP inhibits PI3K-AKT activity via the regulation of PTEN [50,52,53]. However, the absence of arteriovenous (AV) shunts in *Pten*-deficient mouse retina suggests that an increase of PI3K signaling by itself is not sufficient to trigger AVMs. One could speculate that increased PI3K signaling leads to AVMs only in the context of mutation in an AVM causal gene. Inhibition of MAPK-ERK by a MEK inhibitor suppressed ERK phosphorylation and restored localization of vascular ECs cadherin to the junctions of ECs carrying KRAS^G12V^ mutation detected in brain AVM somatic cells. Inhibition of the MAPK-ERK also abrogated the VEGF-like gene signature induced by KRAS^G12V^, but inhibition of the PI3K did not, suggesting that the phenotype of KRAS mutant ECs is specifically mediated by the MAPK-ERK [12]. These findings warrant further investigation to determine the extent to which these signaling pathways are involved in the development and progression of HHT-associated bAVMs.

Genetic mouse models of bAVM have been mostly developed by manipulating *Eng* or *Alk1* genes. *Eng*^+/−^ or *Alk1*^+/−^ heterozygous knockout (KO) mice are viable and show HHT phenotypes during adulthood, while *Eng*^-/-^ or *Alk1*^-/-^ homozygous KO mice are embryonic lethal [38,54,55,56,57,58,59]. bAVMs, including AV shunts and niduses of dilated vessels, occurred in only 30% of *Eng*^+/−^ mice aged 25 to 40 weeks with incomplete penetrance [56]. Brain AVMs did not develop effectively in mice with haploinsufficiency. The hypothesis that a somatic loss of heterozygosity increases AVM formation has been suggested based on animal studies. The deletion of both alleles in either the *Alk1* or *Eng* gene was needed to develop bAVMs [60,61]. *Alk1* deletion derived by the *Alk1* gene promoter resulted in late gestational or postnatal lethality with AVMs in the brain, lung, and intestine, while tamoxifen-induced *Alk1* deletion using R26-CreERT2 in adult mice resulted in AVMs and hemorrhage in visceral organs but not in the brain [62]. Since subdermal vasculatures of *ALK1*-deficient adult mice developed AVMs only in the presence of wounding [62], it was speculated that a secondary angiogenic or inflammatory stimulus would be required for AVM formation in adult brain.

Local VEGF delivery to the brain of *Eng* or *Alk1*-deficient adult mice led to vascular dysplasia, including AV shunting, enlarged tortuous vessels, and micro-hemorrhage [60,63,64,65,66], supporting a critical role of angiogenic stimulus as a secondary insult for de novo bAVM formation in *Eng* or *Alk1*-deficient adult mice. Gross vascular irregularities, including AV shunting and disrupted artery/vein-specific gene expressions, were seen in bAVM lesions in the *Alk1*-model [63].

*Alk1* or *Eng* deficiency in vascular endothelial cells, but not smooth muscle cells, resulted in wound-induced skin AVMs in adult mice [67], while deletion of *Alk1* in ECs, but not in pericytes and macrophages, along with a focal delivery of VEGF led to de novo bAVM formation in adult mice [60,65]. Together, these studies demonstrate that endothelial cells are the primary cells in which ENG-ALK1 signaling regulates for the proper formation of AV networks. Contrary to these studies, *Alk1* or *Eng* deletion using smooth muscle-specific-SM22α-Cre led to the development of bAVMs, including tortuous vessels and hemorrhages in mice [60,61]. Although further investigations are needed to clarify, it is likely that a subset of ECs expressing Cre in this SM22α-Cre line [61] contributes to bAVM development in this model. An interesting study showed that transplantation of ENG-deficient bone marrow (BM) cells caused cerebrovascular dysplasia in wild-type mice after VEGF stimulation [68], suggesting a potential contribution of BM-derived EC progenitors in the pathogenesis of bAVM.

Brain AVMs lesions show high levels of inflammatory signals, including MMP-9 [69] and IL-6 [70]. An abnormally high number of macrophages were present in and around vascular walls in human bAVM specimens, with or without hemorrhage, suggesting that macrophage accumulation is not simply a response to hemorrhage [69,71,72]. Persistent macrophage infiltration and pro-inflammatory differentiation of monocytes in angiogenic tissues contributed to macrophage accumulation in bAVM. In *Alk1-* or *Eng*-deleted bAVM mouse models, the accumulation of macrophages in the brain angiogenic region started between 2 and 4 weeks after angiogenic stimulation before the bAVM formed and incrementally increased up to 8 weeks compared with wild-type mice. More CD34^+^ cells isolated from peripheral blood of HHT patients with either *ENG* or *ALK1* gene mutation differentiated into macrophages than those from healthy controls [73]. However, the deletion of *Eng* in macrophages did not cause AVM formation [60], suggesting that gene deficiency in macrophages is not an initiating factor. The involvement of macrophages might be associated with vascular remodeling and vascular destabilization in bAVM pathogenesis [74,75].

Most of AVM studies to date have been conducted in *Alk1* and *Eng*-deficient mice, but other members of the ENG-ALK1 signaling pathway have been tested to identify whether their deficiency contribute to AVM pathogenesis. Recent reports demonstrated that SMAD4 deficiency led to the development of bAVMs in neonatal mice [50,76]. Matrix Gla protein (MGP), an antagonist of BMPs, is involved in the regulation of VEGF expression by controlling the expression of BMP/TGFβ type I receptors [77]. *Mgp* gene deletion in mice caused AVMs in the brain, enhanced BMP activities, and increased expression of Notch ligands and targets [78]. A recent study showed that Sox2 was elevated in human sporadic bAVM endothelial cells and bAVM endothelial cells of matrix Gla protein null (Mgp^-/-^) mice, causing endothelial-mesenchymal transition (EndMTs) and vessel lumen disruption [79]. Reducing Notch activity by heterozygous deletion of Jagged 1 or 2 prevented bAVM formation [78]. The expression of integrin β8 subunit (ITGB8), interacting with TGF-β signaling, was reduced in sporadic human bAVM [80]. Deletion of Itgb8 enhanced hemorrhage in VEGF-induced brain angiogenic foci of adult *Alk1*^+/−^ mice, suggesting that both Itgb8 and Alk1 are important for maintaining normal cerebral angiogenesis in response to VEGF [81].

A recent report showed that CRISPR/Cas9-mediated somatic *Alk1* gene mutations in wild-type mouse brain induced bAVMs with combined VEGF overexpression [82]. The advantages of this system include that it bypasses the challenges of germline modifications, such as embryonic lethality, and saves the time and cost that are needed for establishing, breeding, and maintaining genetically modified animals. It would especially be useful to generate bAVM models in animals larger than mice.

Some pathogenic variants of genes involved in BMP/TGF-β and VEGF/VEGF receptor (VEGFR) signaling have been detected in DNA extracted from peripheral blood of sporadic bAVM patients. Some of the variants have been implicated to be pathological through testing them in zebrafish [83].

Collectively, these data suggest a specific role of BMP/TGF-β and VEGF/VEGFR signaling in the etiology of bAVM.

## 4. Pdgfb/pdgf Receptor β (pdgfrβ) Pathway

Structural imperfection and immaturity of the vascular wall suggest that bAVM vessels are maldeveloped. Abnormal vessel wall structure has also been noticed in the bAVM vessels in mouse models [63,64]. Compared with normal brain angiogenic foci, the lesions in bAVM mouse models have more vessels with diameters measuring larger than 15 μm and lack smooth muscles.

Blood vessels are composed of endothelial cells and mural cells, including vascular smooth muscle cells and pericytes. Pericytes wrap around the endothelial cells of capillaries and venules. They have a crucial role in vascular stability. The reduction of vascular pericytes impairs vascular integrity [84,85]. Recent studies have shown that both human and mouse bAVM vessels have less mural cell coverage compared to normal brain vessels [64,86,87], suggesting an abnormal vascular remodeling in bAVMs. Reduced vascular smooth muscle cell and pericyte coverage are associated with increased vascular permeability and bAVM hemorrhage [64,86].

PDGF-B and PDGFR-β play an essential role in pericyte- and vascular smooth muscle cell-recruitment during angiogenesis. Knocking out *Pdgfb* or *Pdgfrβ* in mice resulted in the loss of pericytes from the microvessels [88]. The absence of pericytes also led to endothelial hyperplasia and excessive endothelial luminal membrane folds [89]. Abnormal expression of PDGF-B and PDGFR-β has been described in bAVMs in rodent models and patients [86,87,90]. Pdgfβ expression was reduced in the bAVM lesions of *Alk1*-deficient mice [64], which is associated with a reduction of smooth muscle cell- and pericyte-coverage, suggesting a possible link between ALK1 and PDGF-B/PDGFR-β signaling pathways. However, it is not clear whether the reduced expression of PDGFR -β in bAVM is the cause or the result of pericyte reduction. Interestingly, overexpression of PDGF-B in *Alk1*-deficient bAVM increased pericyte coverage on bAVM vessels and reduced bAVM hemorrhage [87].

These data indicate that PDGF-B and PDGFR-β signaling pathway play an important role in bAVM vascular integrity.

## 5. Non-Coding RNA

Recent studies showed that non-coding RNAs might also play a role in bAVM pathogenesis. Li et al. found four long non-coding RNAs were aberrantly expressed in AVM nidus [91]. These non-coding RNAs downregulates nicotinamide adenine dinucleotide phosphate (NADPH) reductase, lipoprotein lipase, etc., which may correlate with seizures in bAVM patients. Through deep sequencing of small RNAs in the blood of patients with bAVM, Chen et al. found novel dysregulated miRNAs, one of which targets the VEGF signaling pathways [92]. It has been observed that miRNA-137 and miRNA-196* inhibit abnormal behavior of AVM smooth muscle cells in culture [93]. In addition, a recent study demonstrated that inactivating mutations in Drosha resulted in vascular abnormalities similar to HHT in mice and angiogenesis defects in zebrafish. DROSHA variants (P100L and R279L) have been detected in HHT patients who lacked known pathogenic mutations [94]. These data indicate that microRNA processing may play a role in AVM pathogenesis.

## 6. Therapeutic Applications

**Figure 1:**
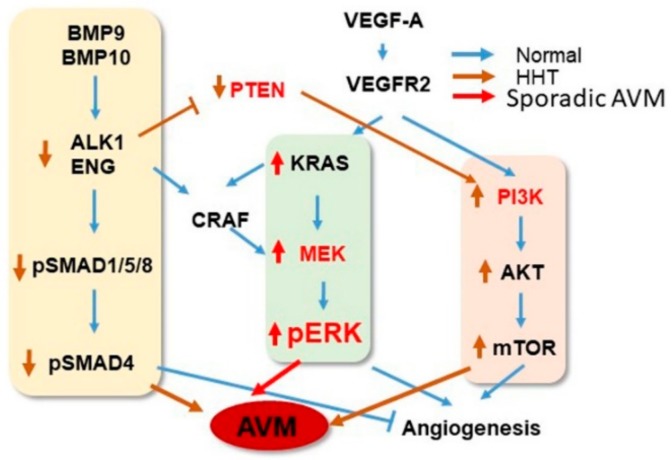
Signaling pathways and therapeutic targets. Normally, BMP9/TGFβ1 regulates angiogenesis through binding to ALK1/ENG to phosphorylate SMAD and increase PTEN activity, which in turn reduces PI3K signaling (blue lines and arrows). BMPs can also signal through MEK/ERK. In HHT (brown lines and arrows), mutation of ALK1 or ENG reduces pSMAD and PETN, resulting in increased PI3K activity or pERK level, causing AVM development. In sporadic AVM cases (red line and arrows), activating mutation in genes in the MAPK-ERK signaling pathway, such as KRAS, BRAF, and MAP2K1, increases the level of pERK, leading to AVM development or progression. The upregulation of PI3K may enhance AVM progression through exacerbation of angiogenesis in HHT. The genes in red can be tested as therapeutic targets.

The signaling pathways for sporadic bAVM and HHT, as well as potential targets for the development of new therapies, are summarized in Figure 1.

In this review, we highlighted the most exciting finding in bAVM research in recent years, including the discovery of somatic mutations in genes in RAS-MAPK pathways in sporadic AVMs [12,13,28,29]. It has been shown that the delivery of MEK inhibitors to AVM ECs reduced ERK activity and decreased vessel abnormalities [12,29]. The existing FDA approved inhibitors for the ERK pathway (https://www.cancer.gov/about-cancer/treatment/drugs/fda-trametinib) can accelerate the translation of the knowledge obtained from this study to clinical practice.

Elevated VEGF expression in human bAVMs has been implicated in bAVM pathophysiology [95,96,97] and bAVM hemorrhage in a mouse bAVM model with *Alk1* deletion [98]. VEGF antagonism by bevacizumab, a humanized monoclonal antibody neutralizing all VEGF-A isoforms, reduced the number of dysplastic vessels in the bAVM model in which *Alk1* was deleted, and VEGF was virally overexpressed [99]. Soluble FMS-like tyrosine kinase 1 (sFLT1) contains only the extracellular domains of FLT1 (VEGFR1) and binds with VEGF strongly [100]. Intravenously delivered adeno-associated viral vector serotype-9, expressing sFLT1 (AAV9-sFLT1), reduced bAVM severity in two bAVM models; model 1 where *Eng* was deleted globally, and VEGF was virally overexpressed in the brain focally, and model 2 where *Eng* was deleted by SM22α-Cre [101]. Therefore, inhibition of VEGF downstream signaling could be an effective strategy to reduce bAVM severity.

Thalidomide reduces nose bleeding and stimulates vessel maturation in HHT patients and improves mural cell recruitment in the retina of *Eng*^+/−^ mice [102]. Thalidomide, or its less toxic analog, lenalidomide, treatment increased pericyte coverage of bAVM vessels and reduced the number of dysplastic vessels and hemorrhage in the bAVM lesion in the adult-onset bAVM mouse model [87]. Therefore, thalidomide or lenalidomide could also be a therapeutic option to stabilize bAVM vessels and prevent bAVM rupture.

Currently, there is no medical therapy for bAVMs. Bevacizumab, mitogen-activated protein kinase enzyme (MEK) inhibitors, rapamycin, and thalidomide have all been used with varying degrees of efficacy for patients with non-central nervous system AVMs. The most devastating symptom of bAVM is ICH, unlike cancer-related chemotherapy that aims to shrink abnormal tumor tissue. The concept for the treatment of bAVM should be to stabilize vascular tissue and thereby decrease the risk of spontaneous ICH. Therefore, in addition to identifying the pathways that are involved in the development of AVMs, understanding the mechanisms and factors involved in vascular remodeling, maintenance, vascular integrity, and rupture of AVMs is crucial for developing strategies to stabilize the AVM vessel wall and prevent AVM rupture.

## Abbreviations

bAVM	Brain arteriovenous malformation
HHT	hereditary hemorrhagic telangiectasia
TGFβ	transforming growth factor β
Pdgfb	platelet-derived growth factor b
ENG	endoglin
ALK1 or ACVLR1	activin receptor-like kinase 1
